# In Situ Degradation Kinetics of 25 Feedstuffs and the Selection of Time Points in Mathematical Statistics

**DOI:** 10.3390/ani13050947

**Published:** 2023-03-06

**Authors:** Sen Li, Liwen He, Fang Mo, Wei Zhang

**Affiliations:** State Key Laboratory of Animal Nutrition, College of Animal Science and Technology, China Agricultural University, Beijing 100193, China

**Keywords:** nylon bag technology, rumen degradation, sheep, simplified method, time points selection

## Abstract

**Simple Summary:**

In order to further standardize and simplify the operation of the nylon bag method, rumen degradation kinetics of 25 feedstuffs were first determined using in situ nylon bag technique and then the differences of degradation parameters fitted with five or seven time points measuring data were evaluated. Additionally, the goodness of fit (R^2^) of degradation curves obtained at different time points was compared. In protein feeds, energy feeds and roughages, there were no significant differences in rumen degradation parameters except for several feedstuffs. By comparing the R^2^, it was found that the R^2^ of degradation curves which obtained five time points was better than that which obtained seven time points. These results indicate that it is feasible to determine the rumen degradation characteristics of feedstuffs by only setting five measuring time points, and based on the reasonable criteria, where the optimal selection of incubation time points for protein feeds and energy feeds was the set of ② 2, 16, 24, 36, 48 h, and that for roughages was ① 4, 8, 16, 48, 72 h.

**Abstract:**

Rumen degradation kinetics of 25 feedstuffs (six protein feeds, nine energy feeds and ten roughages) were first determined using the nylon bag technique in situ and the differences of degradation characteristics fitted with five or seven time points measuring data were evaluated with the goodness of fit (R^2^) of degradation curves. Protein and energy feeds were incubated for 2, 4, 8, 16, 24, 36, 48 h, roughages were incubated for 4, 8, 16, 24, 36, 48, 72 h, where three and six data sets of five time points were screened out, respectively. Only the degradation parameters a (rapidly degraded proportion), b (slowly degraded proportion) and c (degradation rate of slowly degraded proportion) of several feeds at five time points were significantly different from those at seven time points (*p* < 0.05), and the others were not significant (*p* > 0.05). The R^2^ of the degradation curves obtained at five time points was closer to 1, indicating that the fitting obtained at five time points was more accurate in predicting the real-time rumen degradation rate of feed. These results indicate that it is feasible to determine the rumen degradation characteristics of feedstuffs by only setting five measuring time points.

## 1. Introduction

Though the nylon bag technique has been used extensively for evaluating the rumen degradation profile of feedstuffs, discrepancies are commonly found between the studies deriving from inter- and intralaboratory on the aspect of measurement procedures. The difference in any factor of bag size, feed particle and amount, sampling rule, times of sampling, washing method, and mathematical calculation would likely affect the results. To enhance the comparability of the results from different studies and assure the reliability of the measurement, standard procedures were recommended and improved more than once [1,2].

However, the incubation times recommended procedures which might remarkably influence the accuracy and efficiency of the evaluation. Ørskov [3] suggested that concentrates should be cultured in rumen for 2, 6, 12, 24 and 36 h; Lindberg [4] recommended concentrates for 2, 4, 8, 16 and 24 h, and roughages for 2, 4, 8, 16, 24, 36, 48 h; AFRC [1] recommended that concentrates should be cultured for 2, 6, 8, 24, 48 h, and roughages for 2, 6, 8, 24, 48, 72 h. Michalet-Doreau [5] and Vanzant [6] suggested that the required number of time points should be able to describe the curve. The recent studies that used the nylon bag method to determine rumen degradation characteristics of feedstuff on sheep are briefly summarized in Table 1. It is evident that various numbers and hours of sampling were used in different studies, with the number of sampling time point being mostly in the range of 5–9 and the longest incubation duration being 72 h in most research, such as A.R. Seradj [7], L. Tao [8], B. Ghorbani [9], Xiaogao Diao [10], and so on [11,12,13,14,15,16]. Systematic studies are in need to make clear the influence of sampling hours and sampling times on the results of the rumen nylon bag technique.

In this study, rumen degradation characteristics of 25 feeds (six protein feeds, nine energy feeds, and ten roughages) were determined on fistulated sheep by the nylon bag technique with the setting of seven sampling time points. The degradation parameters a, b, c obtained at five time points were compared with those obtained at seven time points. Furthermore, the R^2^ of degradation curve estimated with five or seven time points were compared, to investigate the feasibility of reducing the number of sampling time point in the measurement of rumen degradation parameters of feedstuffs. The outcome of this study would enrich the database of nutritive value of feedstuffs in sheep, and provide a reference for the application of the nylon bag technique. 

## 2. Materials and Methods

All animal management and experimental procedures followed the animal care protocols approved by the Animal Care and Use Ethics Committee of China Agricultural University.

### 2.1. Animals and Diets

Eight ruminally fistulated Wether sheep aged 2 to 3 years old with an average live weight of 57.4 ± 2.4 kg were selected and divided into two groups. They were then placed into a group of 4 sheep (i.e., four replicates) to determine rumen degradation parameters of different feeds. The animals were fed twice daily at 8:00 and 17:30, with free access to clean water. Sheep were fed a ration (DM basis) consisting of 45.00% soybean stem, 25.00% wheat straw, 18.56% corn, 4.95% soybean meal, 4.95% wheat bran, 0.62% CaHPO_4_, 0.31% NaCl, 0.31% sodium bicarbonate, and 0.3% premix.

### 2.2. Samples Preparation and Nutrient Analysis

A total of 25 feedstuffs collected around the country were used in the present study and the nutritional compositions are presented in Table 2. To prepare feed samples, raw materials were dried at 65 °C for 48 h in a forced-air oven and then milled through a 1 mm sieve for chemical analysis and 2.5 mm sieve for in situ degradation. The concentrations of dry matter (DM), organic matter (OM), and fat were analyzed according to the methods of AOAC [17]. Nitrogen (N) content was measured by the Kjeldahl method [17] using a FOSS semi-automatic nitrogen analyzer, and crude protein (CP) content was calculated as N × 6.25. The contents of neutral detergent fiber (NDF) and acid detergent fiber (ADF) were analyzed using an automatic fiber analyzer (A2000i, Ankom Technology, Macedon, NY, USA) following the methods described by Van Soest et al. [18].

### 2.3. In Situ Nylon Bag Experiment

The in situ degradabilities of DM, CP, and OM in the 25 feeds were determined according to the procedure described by Mehrez and Ørskov [19]. A given amount of feed sample, i.e., 3 g for protein feeds, 3 g for energy feeds, or 5 g for roughages, was weighed into the nylon bag (48 μm pore size, 6 cm × 10 cm bag size) in duplicate, 1 feed was cultured in rumen of each sheep and a total of 14 nylon bags. The tied bags were placed into the rumen before the morning feeding at 0800 and removed at the given time points. Differently, the incubation time points for protein feeds or energy feeds were set as 2, 4, 8, 16, 24, 36, 48 h, while the roughages were incubated for 4, 8, 16, 24, 36, 48, 72 h. After the removal from the rumen, the bags were immediately washed under running water till the flow-out water was clear, in order to stop microbial fermentation [20]. Then, the bags with clean residue were dried to a constant weight at 65 °C for 48 h and weighed. The residues were further ground through a 1 mm sieve for nutrient analysis.

### 2.4. Calculations of Degradation Kinetics Parameters

The kinetic parameters of in situ degradation were calculated based on the measured degradabilities at all 7 time points or 5 selective time points. The data of instant degradability were fitted using the following exponential equation:

Y = a + b (1 − e^−ct^) (1)

where Y is the nutrient disappearance at time point t, a is the rapidly degradable fraction, b is the potentially degradable fraction, c is the degradation rate of fraction b (%/h), and t is the time (h) of incubation.

ED = a + bc/(c + k)(2)

where a, b, and c are the same as those in Equation (1), and k is the rumen outflow rate. In this study, the rumen outflow rate was set by referring to previous studies, i.e., roughages 3.14%/h [21], DDGS 3.99%/h, silage feeds 2.53%/h, and oil-seed-meals 5%/h [22]. To compare the difference of the degradation kinetic parameters deriving from the calculation with 5 time points or 7 time points data, the potential combinations of 5 time points were screened out as follows: ① 2, 16, 24, 36, 48 h, ② 2, 8, 16, 24, 48 h, ③ 2, 8, 16, 36, 48 h for protein feeds and energy feeds, and ① 4, 16, 36, 48, 72 h, ② 4, 16, 24, 48, 72 h, ③ 4, 16, 24, 36, 72 h, ④ 4, 8, 16, 24, 72 h, ⑤ 4, 8, 16, 36, 72 h, ⑥ 4, 8, 16, 48, 72 h for roughages. The selection was as follows: by referring to the relevant literature and observing the rumen degradation rate curve, it was found that the longest incubation time of concentrates and roughages in the rumen were 48 h and 72 h, respectively. At this time, the degradation curve tended to be flat. The rumen degradation rate of 16 h could be used to calculate the small intestine digestibility of feeds. Considering the properties of the feeds, the degradation rate of protein and energy feeds in the rumen was faster, while that of roughages was slower. In addition, according to the research basis of our laboratory, 2 h and 4 h were selected as the shortest culture time of protein/energy feeds and roughages, respectively. Therefore, the shortest and longest time points, and 16 h of feed culture in the rumen, were kept. In addition, in order to reduce the stress caused by excessive density of time points, the protein/energy feeds culture for 4 h were removed.

The degradation parameters “a”, “b”, and “c” were calculated at 5 time points in the same way as Formula (1).

### 2.5. Reasonable Criteria for Selection

The criteria for selecting the optimal combination were as follows, ① when the degradation parameters (a, b, c) obtained at 5 time points were closer to the values obtained at 7 time points (the difference was not significant), it indicated that the selected time point combination was the best; ② when the R^2^ of fitting curve of DM, CP, and OM obtained from 5 time points was closer to 1, it indicated that the combination was the best.

### 2.6. Statistical Analyses

The data concerning nutrients disappearance and kinetic parameters a, b, and c were analyzed using the general linear model (GLM) procedure of SAS. The differences of degradation parameters were calculated with SPSS. Difference was considered significant when *p* < 0.05.

## 3. Results

### 3.1. The Parameters a, b, c and ED of DM, CP and OM of the Feedstuff

The parameters a, b, c and ED of 25 feeds are summarized in Table 3, Table 4 and Table 5. In general, the a, b, c and ED of DM, OM, and CP of each feedstuff obtained at five time points were different from those obtained at seven time points (*p* < 0.05).

For the protein feeds, the “a” of CP and OM of CSM, the “b” of CP, and the “a” of OM of CGM, the “a” of DM of DDGS, and the “b” of CP of SOM obtained at five time points of ①, ②, ③, ④ were significantly different from those obtained at seven time points (*p* < 0.05). The ED of CP of CGM and ES obtained at five time points of ①, ②, ③, ④ were significantly different from those obtained at seven time points (*p* < 0.05). There were no significant differences in other degradation parameters (*p* > 0.05).

For energy feeds, the “a” and “b” of DM, CP, and OM of BY and WT, the “b” of DM of HS, the “a” and “b” of DM of HY9 and GWC, the “a” of DM of YC, and the “b” of DM of YWB obtained at five time points of ①, ②, ③, ④ were significantly different from those obtained at five time points (*p* < 0.05). The ED of DM, CP, and OM of CBS, the ED of DM of YWB and GWC obtained at five time points of ①, ②, ③, ④ were significantly different from those obtained at seven time points (*p* < 0.05). There were no significant differences in other degradation parameters (*p* > 0.05).

For roughages, the “a” and “b” of CP of RG, the “a” and “b” of DM of RSW, the “a”, “b”, and “c” of OM of RSW and TP, the “b” of DM of OG and CS, the “c” of OM of CS, the “a” of DM of TP, and the “a”, “b”, and “c” of CP of TP obtained at five time points of ①, ②, ③, ④, ⑤, ⑥ were significantly different from those obtained at seven time points (*p* < 0.05). The ED of DM and OM of RSW and the ED of DM, CP, and OM of TP obtained at five time points of ①, ②, ③, ④, ⑤, ⑥ were significantly different from those obtained at seven time points (*p* < 0.05). There were no significant differences in other degradation parameters (*p* > 0.05).

### 3.2. The R^2^ of Fitted Curves of DM, CP, and OM Obtained at Different Time Points

**Figure 1:**
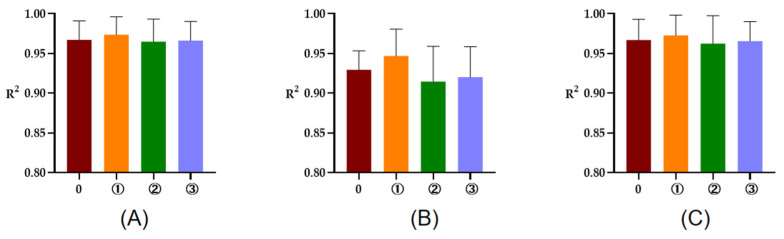
The R^2^ of fitted curve of DM, CP, and OM of protein feeds of 5 or 7 time points. (**A**–**C**), respectively, represent the R^2^ of fitted curve of DM, CP, and OM in the rumen of protein feeds. The number 0 = 7 incubation time points, ①, respectively, the R^2^ of fitted curve of 2, 16, 24, 36, 48 h; ②, respectively, the R^2^ of fitted curve of 2, 8, 16, 24, 48 h; ③, respectively, the R^2^ of fitted curve of 2, 8, 16, 36, 48 h.

**Figure 2:**
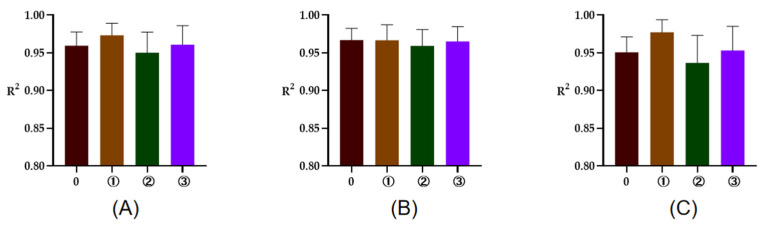
The R^2^ of fitted curve of DM, CP, and OM of energy feeds of 5 or 7 time points. (**A**–**C**), respectively, represent the R^2^ of fitted curve of DM, CP, and OM in the rumen of energy feeds. The number 0 = 7 incubation time points, ①, respectively, the R^2^ of fitted curve of 2, 16, 24, 36, 48 h; ②, respectively, the R^2^ of fitted curve of 2, 8, 16, 24, 48 h; ③, respectively, the R^2^ of fitted curve of 2, 8, 16, 36, 48 h.

**Figure 3:**
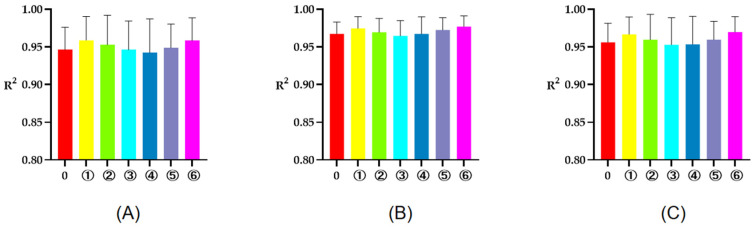
The R^2^ of fitted curve of DM, CP and OM of roughages of 5 or 7 time points. (**A**–**C**), respectively, represent the R^2^ of fitted curve of DM, CP, and OM in the rumen of roughages. The number 0 = 7 incubation time points, ①, respectively, the R^2^ of fitted curve of 4, 16, 36, 48, 72 h; ②, respectively, the R^2^ of fitted curve of 4, 16, 24, 48, 72 h; ③, respectively, the R^2^ of fitted curve of 4, 16, 24, 36, 72 h; ④, respectively, the R^2^ of fitted curve of 4, 8, 16, 24, 72 h; ⑤, respectively, the R^2^ of fitted curve of 4, 8, 16, 36, 72 h; ⑥, respectively, the R^2^ of fitted curve of 4, 8, 16, 48, 72 h.

The R^2^ of rumen degradation fitted curves of DM, CP, and OM in the protein feeds, energy feeds, and roughages of 5 or 7 time points are illustrated in Figure 1, Figure 2 and Figure 3. As seen in the figures, the R^2^ of degradation curves fitted with 5 or 7 time points of each feed was greater than 0.9. Additionally, the R^2^ of ① 2, 16, 24, 36, 48 h was closer to 1 for protein and energy feeds, the R^2^ of ⑥ 4, 8, 16, 48, 72 h was closer to 1. The closer the value of R^2^ was to 1, the better the fitted curve fit the observed value (rumen degradation rate of feeds).

## 4. Discussion

The degradation parameters of various types of feeds were different in the rumen, and the effect of different time points combination (①,② and ③ of protein feeds and energy feeds, ①, ②, ③, ④, ⑤, and ⑥ of roughages) on the “a”, “b”, “c” and ED of feeds was also different. The rate and extent of DM fermentation in the rumen were important determinants of the degree of ruminal digestion [23], and they could be affected by the factors of processing, feed property, animal physiological status, etc.

For protein feeds, compared with the degradation parameters “a”, “b”, and “c” of DM and OM, the degradation parameters of CP at five time points had a greater effect, which may have been related to the structure of protein in feeds. For energy feeds, compared with the parameters obtained at seven time points, the effects of different combinations of five time points on degradation parameters were different. Roughages generally have higher NDF and ADF contents. The main components of cell wall NDF and ADF exerted a dramatical limiting effect on the digestibility of forage. For TP, the “a” of DM, the “a” and “c” of CP and OM at five time points (①, ②, ③, ④, ⑤ and ⑥) were significantly lower than that at seven time points; the ED of DM, CP, and OM were significantly higher than that at seven time points. Five time points may not have been suitable to evaluate TP, and the specific reasons need to be further studied. In conclusion, using five time points to evaluate rumen degradation characteristics will lead to changes in degradation parameters (“a”, “b”, and ”c”), but has little effect on ED.

Feed nutrients mainly include rapidly degraded proportion (a), slowly degraded proportion (b), and unstable proportion. The changes of a, b, and c would affect the ED. Although the ED was affected by outflow rate, for this study, the base diet of the sheep was consistent; thus, the effect of fewer samples in nylon bags on outflow rate was negligible. Additionally, ED was one of the most important data points for evaluating the nylon bag method and should be retained. By comparing the degradation parameters obtained at five or seven time points, it was found that the selection of different time points would lead to significant differences in a, b, and c, but it did not show in ED, which may have been because the influence of different time points on the a and b of the feed was cancelled out in the calculation of ED. 

The nylon bag technique is a common method to evaluate the nutritional value of ruminant feeds, but the standardization of its measurement procedure needs further improvement. Generally, the more time points measured, the more accurate degradation parameters and ED obtained, and the higher fitting degree of degradation curve obtained. The dynamic degradation curve would evidently present the fermentation characteristics of the feeds in the rumen. Michalet-Doreau [5] and Vanzant [6] suggested that the number of time points could be used to describe the curve. The British AFRC [1] recommended that the nylon bag method be used to determine the rumen degradation characteristics of concentrate and roughage at five time points. In the present study, the rumen degradation curve of nutrients could be obtained at five time points and little gap between the curves fitted with five or seven time points data were found, suggesting a strong feasibility of the simplification of time points in the nylon bag technique. In addition, given that too many time points will much increase workload and cause serious stress to the experimental animals, animal welfare in experimental protocols would be improved and the cost and labor would be reduced by reducing measurement time points. On the other hand, setting too many time points with short time intervals would likely affect the normal function of the rumen and, consequently, affect the test results, in that frequent extraction and placement would lead to the long-time exposure of rumen microbes to the external environment.

In this study, it was feasible to use five time points to calculate the degradation parameters “a”, “b”, and “c” of feeds, and according to the reasonable selection criteria (the difference was not significant, the degradation parameters were closer to seven time points, and the R^2^ of fitted curve was closer to 1), for protein and energy feeds, the degradation parameters obtained by using ① 2, 16, 24, 36, 48 h were closer to those obtained by using seven time points, and the R^2^ of the fitting curve obtained was better than the seven time points, for roughages, the degradation parameters obtained by using ⑥ 4, 8, 16, 48, 72 h were closer to those obtained by using seven time points, and the R^2^ of the fitting curve obtained was better than the seven time points.

## 5. Conclusions

The results of this study showed that rumen degradation parameters (“a”, “b”, and “c”) varied with different time points, but had little effect on ED, and the R^2^ of fitted curves obtained five time points was closer to 1. It is feasible to determine the rumen degradation characteristics of feedstuff at five time points. Moreover, the optimal combinations of five rumen incubation time points were found to be 2, 16, 24, 36, 48 h for protein and energy feeds, and 4, 8, 16, 48, 72 h for roughages.

## Figures and Tables

**Table 1:** Comparison of recent studies on the application of nylon bag technique for rumen degradability in sheep.

Items			Bag				Repliacation		Incubation Conditions	
	Diet	Feeding Level	Material	Pore Size	Sample Grind Size	Animal	Number of Animals	Number of Bags	Number of Times	Times
A.R.Seradj [7]	—	ad libitum	Dacron bags	45 μm	—	Rasa Aragonesa ewes	4	1	5	4, 8, 12, 24, 48 h (Moringa oleifera)
L.Tao [8]	—	—	—	—	—	Hancrossbred ewes	6	—	5	6, 12, 24, 48, 72 h (roughages)
B.Ghorbani [9]	—	maintenance level	polyamide	40 ± 10 μm	—	Zel ewes	3	4	—	1, 3, 6, 12, 24, 36, 48, 72, 96 h (sesame meal, soybean meal)
Xiaogao Diao [10]	70:30	maintenance	nylon cloth	48 μm	2.5 mm (roughages)	Yunnan semi-fine wool sheep	4	2	7	2, 4, 8, 16, 24, 36, 48 h (72 for roughages)
S.Rjiba-Ktita [11]	—	ad libitum	—	50 μm	3 mm	Barbarine rams	4	2	6	3, 6, 24, 48, 72, 96 (seagrass, macroalgae, barley grain and barley grass)
M.A.Harahap [12]	—	maintenance level	—	46 μm	—	local Ettawah cross bred goats	2	3	7	0.75, 1.5, 3, 6, 12, 24, 48
V.Palangi [13]	60:40	—	polyester mesh bag	47 μm	2 mm	Ghezel sheep	3	2	7	0, 4, 8, 16, 24, 36, 48 h (barley)
Mohammad Farhad Vahidi [14]	70:30	ad libitum	heat-sealed nylon bag	50 μm	2 mm	Shal sheep	3	2	5	0, 24, 48, 72, 96 h (lignocellulosic forages)
Biwei Jiang [15]	—	ad libitum	—	—	40 mesh (roughages)	tan sheep	3	2	7	3, 6, 12, 24, 36, 48, 72 h (roughages)
Rodrigo A.C. Passetti [16]	—	—	R1020	50 μm	4 mm (roughages)	ewe lambs	3	—	9	1, 3, 6, 12, 24, 36, 48, 72, 120 h (roughages)

— means no date information was involved.

**Table 2:** Routine nutrients in common feeds for sheep (DM basis).

Items		OM (%)	CP (%)	Fat (%)	ADF (%)	NDF (%)
	RSM	92.44	38.01	11.56	44.85	58.88
	CSM	93.04	50.01	0.70	12.08	22.32
protein feeds	CGM	98.88	65.05	0.59	10.33	40.21
	ES	94.66	39.62	23.51	11.88	24.63
	DDGS	95.02	28.70	10.85	18.00	41.83
	SOM	93.18	49.77	1.60	10.38	18.68
	BR	99.12	12.42	1.12	2.19	4.69
	BY	98.62	13.84	0.71	3.70	27.55
	WT	98.02	13.45	1.52	3.65	12.73
	HS	98.39	10.92	2.91	6.40	14.56
energy feeds	HY9	98.78	8.17	3.05	4.65	12.14
	GWC	98.24	10.44	4.93	1.22	7.72
	YC	98.60	9.31	3.16	3.41	9.69
	YWB	94.03	17.95	3.71	13.92	44.61
	CBS	96.43	18.68	1.98	17.32	59.80
	WS	91.75	9.31	2.13	40.07	70.26
	RG	88.12	9.90	1.73	33.48	56.80
	RSW	86.62	12.44	1.43	33.47	45.84
	BS	91.24	14.33	2.36	32.61	52.69
roughages	SS	89.88	12.87	1.44	35.21	48.35
	CWR	93.08	10.15	0.70	44.66	72.33
	RS	94.91	15.99	2.57	38.99	66.39
	OG	88.93	16.92	1.85	34.82	63.96
	CS	95.46	10.33	3.42	27.35	47.13
	TP	92.32	17.23	0.91	19.08	26.93

**Table 3:** The degradation parameters of dry matter, crude protein, and organic matter in rumen of six protein feeds (%).

		DM				CP				OM			
		a	b	c	ED	a	b	c	ED	a	b	c	ED
	**0**	24.31	29.34	0.055	39.39	9.75	40.12 ^ab^	0.089 ^a^	35.11	22.44	31.48	0.054 ^b^	39.93
RSM	①	22.70	31.51	0.050	38.52	11.83	41.31 ^a^	0.057 ^b^	33.89	20.44	30.24	0.089 ^a^	39.81
	②	23.24	30.38	0.056	39.22	12.13	37.48 ^b^	0.087 ^a^	35.92	20.68	31.59	0.080 ^a^	40.07
	③	23.48	29.62	0.055	39.00	10.59	38.65 ^ab^	0.100 ^a^	36.31	20.10	30.83	0.090 ^a^	39.91
	**0**	15.44	54.74	0.064	46.12	10.94 ^c^	66.86	0.056 ^a^	46.02	13.21 ^b^	54.03	0.072	45.10
CSM	①	16.60	54.33	0.060	46.23	16.32 ^a^	66.82	0.042 ^b^	46.75	16.95 ^a^	53.30	0.058	45.62
	②	16.50	53.73	0.060	45.81	16.59 ^a^	64.53	0.042 ^b^	45.93	16.94 ^a^	52.53	0.059	45.31
	③	15.94	55.10	0.061	46.29	14.47 ^b^	66.28	0.049 ^ab^	47.34	16.37 ^a^	53.79	0.060	45.78
	**0**	16.72 ^a^	39.79	0.076	42.67	8.58 ^a^	67.30 ^a^	0.023	42.67 ^a^	16.82 ^a^	40.98	0.072	42.98
CGM	①	14.07 ^ab^	41.59	0.084	42.23	8.84 ^ab^	63.78 ^b^	0.024	32.79 ^b^	14.35 ^b^	42.49	0.078	42.51
	②	13.99 ^ab^	41.12	0.091	42.60	7.91 ^b^	66.22 ^ab^	0.024	32.72 ^b^	14.42 ^b^	42.08	0.084	42.98
	③	13.67 ^b^	41.53	0.093	42.76	10.15 ^a^	52.16 ^c^	0.033	33.64 ^b^	14.19 ^b^	42.00	0.087	42.95
	**0**	25.71	68.69	0.043	60.62	16.81	67.72 ^a^	0.034	60.62 ^a^	24.23	67.95 ^a^	0.047	57.46
ES	①	23.81	68.53	0.046	56.68	16.37	68.29 ^a^	0.032	43.17 ^b^	23.79	67.12 ^ab^	0.049	56.94
	②	24.13	66.27	0.047	56.17	17.72	63.71 ^b^	0.033	42.86 ^b^	23.80	65.63 ^b^	0.049	56.22
	③	23.58	68.48	0.047	56.76	16.35	64.64 ^b^	0.037	43.84 ^b^	23.49	67.98 ^a^	0.048	56.68
	**0**	26.44 ^b^	50.96 ^a^	0.039	50.90	12.35	38.83 ^b^	0.036	30.79	24.74 ^bc^	57.60 ^a^	0.037	50.73 ^ab^
DDGS	①	29.45 ^a^	50.19 ^ab^	0.031	51.55	13.07	36.07 ^c^	0.040	31.17	29.45 ^a^	50.19 ^bc^	0.031	51.55 ^a^
	②	29.56 ^a^	49.70 ^ab^	0.030	50.77	12.34	37.30 ^bc^	0.037	30.34	26.46 ^b^	49.03 ^c^	0.035	49.44 ^b^
	③	27.80 ^ab^	47.88 ^c^	0.040	51.83	12.93	41.88 ^a^	0.028	30.31	23.72 ^c^	51.28 ^b^	0.047	51.54 ^a^
	**0**	30.45	63.41 ^a^	0.037	57.23	14.23 ^ab^	76.13 ^b^	0.032	43.63	29.48	64.83	0.033	55.28
SOM	①	30.60	62.65 ^ab^	0.037	57.33	16.08 ^a^	70.67 ^c^	0.034	44.68	28.97	65.33	0.029	55.09
	②	30.79	61.38 ^b^	0.037	56.81	13.88 ^b^	77.35 ^b^	0.032	43.95	29.29	64.19	0.033	55.00
	③	29.85	62.08 ^ab^	0.040	57.44	15.30 ^ab^	82.39 ^a^	0.026	43.20	28.70	63.55	0.036	55.35

^a–c^ indicate that there are significant differences of ED of the same feed in the same column (*p* < 0.05). The same applies in the following tables. 0 respectively 7 incubation time points; ① respectively 2, 16, 24, 36, 48 h; ② respectively 2, 8, 16, 24, 48 h, ③ respectively 2, 8, 16, 36, 48 h.

**Table 4:** The degradation parameters of dry matter, crude protein, and organic matter in rumen of nine energy feeds (%).

		DM				CP				OM			
		a	b	c	ED	a	b	c	ED	a	b	c	ED
	**0**	13.13 ^a^	78.86 ^ab^	0.046	50.97	9.86 ^a^	81.21 ^b^	0.045	48.46	13.34 ^a^	79.31	0.045	50.69
BR	①	11.77 ^ab^	78.02 ^b^	0.050	50.94	8.07 ^ab^	81.98 ^ab^	0.048	48.01	11.65 ^ab^	79.18	0.048	50.43
	②	11.00 ^b^	80.29 ^a^	0.048	50.37	7.93 ^b^	81.97 ^ab^	0.047	47.73	11.22 ^b^	79.77	0.047	49.79
	③	10.17 ^b^	80.00 ^a^	0.051	50.72	8.78 ^ab^	83.35 ^a^	0.043	47.41	10.62 ^b^	80.59	0.048	50.22
	**0**	33.95 ^a^	62.97 ^c^	0.086	73.62 ^a^	18.2 ^a^	74.88 ^c^	0.061	58.84	33.86 ^a^	63.12 ^c^	0.088	73.83 ^a^
BY	①	27.90 ^c^	67.06 ^b^	0.109	73.91 ^a^	14.87 ^b^	78.89 ^a^	0.058	57.30	27.68 ^b^	67.34 ^b^	0.111	74.13 ^a^
	②	28.76 ^c^	68.66 ^ab^	0.085	72.05 ^ab^	15.47 ^b^	76.71 ^b^	0.062	57.90	28.67 ^b^	68.81 ^ab^	0.086	72.24 ^ab^
	③	28.10 ^c^	69.45^a^	0.088	72.35 ^a^	14.8 ^b^	77.38 ^ab^	0.064	58.27	28.00 ^b^	69.64 ^a^	0.089	72.55 ^a^
	**0**	37.43 ^a^	55.96 ^c^	0.089	73.23	20.81 ^a^	73.66 ^d^	0.078 ^ab^	65.48 ^a^	40.14 ^a^	51.76 ^d^	0.102 ^a^	74.75 ^a^
WT	①	34.18 ^b^	60.60 ^a^	0.079	71.36	14.90 ^b^	82.51 ^a^	0.068 ^b^	62.30 ^b^	34.32 ^b^	60.42 ^a^	0.079 ^c^	71.27 ^c^
	②	34.36 ^b^	60.10 ^a^	0.087	72.46	15.61 ^b^	79.91 ^b^	0.077 ^ab^	64.03 ^ab^	34.18 ^b^	58.86 ^bc^	0.095 ^ab^	72.70 ^bc^
	③	35.08 ^b^	58.22 ^b^	0.086	71.82	15.69 ^b^	79.05b ^c^	0.078 ^ab^	63.79 ^ab^	34.43 ^b^	58.20 ^c^	0.094 ^ab^	72.48 ^bc^
	**0**	9.73 ^b^	83.32 ^b^	0.040	46.48 ^b^	11.67 ^a^	78.98 ^b^	0.041	46.43 ^ab^	14.98 ^b^	80.31 ^a^	0.037	49.12
HS	①	10.22 ^a^	77.75 ^c^	0.049	48.50 ^a^	8.00 ^c^	83.41 ^a^	0.039	44.71 ^b^	17.92 ^a^	73.07 ^b^	0.041	50.84
	②	7.48 ^b^	86.21 ^a^	0.043	47.14 ^ab^	9.97 ^ab^	77.21 ^b^	0.043	45.53 ^ab^	15.28 ^b^	80.87 ^a^	0.037	49.40
	③	8.75 ^ab^	86.00 ^a^	0.039	46.33 ^b^	9.84 ^ab^	78.62 ^b^	0.042	45.82 ^ab^	15.53 ^b^	80.38 ^a^	0.036	49.23
	**0**	16.39 ^a^	79.85 ^a^	0.036 ^ab^	49.77 ^a^	23.28 ^a^	72.2 ^ab^	0.026	47.76	12.80 ^b^	71.24 ^ab^	0.055 ^ab^	50.14 ^bc^
HY9	①	14.36 ^bc^	72.30 ^c^	0.043 ^ab^	47.71 ^bc^	21.11 ^b^	73.8 ^a^	0.026	46.48	15.47 ^a^	63.45 ^d^	0.072 ^a^	52.96 ^a^
	②	13.55 ^cd^	74.14 ^b^	0.041 ^ab^	47.04 ^bc^	21.74 ^ab^	71.59 ^b^	0.027	47.02	12.96 ^b^	72.66 ^a^	0.054 ^ab^	50.69 ^b^
	③	15.61 ^ab^	80.55 ^a^	0.030 ^b^	46.07 ^c^	21.33 ^b^	67.9 ^c^	0.031	47.11	13.35 ^b^	70.72 ^c^	0.054 ^ab^	50.20 ^bc^
	**0**	36.72 ^a^	57.9 ^a^	0.038	61.80 ^a^	31.42	51.57 ^c^	0.040	54.15	27.28 ^ab^	65.48 ^b^	0.044 ^a^	57.54 ^ab^
GWC	①	29.67 ^b^	53.37 ^b^	0.051	56.67 ^b^	29.78	55.06 ^a^	0.037	53.23	26.13 ^b^	66.57 ^b^	0.043 ^a^	57.06 ^ab^
	②	30.29 ^b^	50.97 ^c^	0.055	56.97 ^b^	30.07	54.48 ^ab^	0.038	53.67	26.41 ^b^	66.03 ^b^	0.044 ^a^	57.35 ^ab^
	③	29.73 ^b^	51.88 ^bc^	0.057	57.37 ^b^	29.71	52.87 ^bc^	0.042	53.72	28.46 ^a^	69.73 ^a^	0.033 ^b^	56.39 ^b^
	**0**	29.07 ^a^	57.05 ^b^	0.033	51.62	27.70	30.84	0.057 ^a^	44.03	26.51	71.58 ^a^	0.033	54.57
YC	①	25.79 ^b^	60.54 ^a^	0.034	50.21	27.52	31.07	0.056 ^a^	43.91	24.77	71.77 ^a^	0.034	53.92
	②	27.12 ^b^	56.58 ^b^	0.036	50.96	27.59	31.08	0.056 ^a^	44.05	25.00	71.22 ^ab^	0.035	54.23
	③	26.91 ^b^	56.34 ^b^	0.038	51.09	28.57	30.90	0.047 ^b^	43.54	24.71	69.61 ^b^	0.037	54.27
	**0**	32.86	43.82 ^b^	0.053 ^ab^	77.20 ^a^	29.42 ^a^	58.34 ^b^	0.151	75.70	25.85	44.2 ^ab^	0.077 ^ab^	54.54
	①	32.30	46.91 ^a^	0.038 ^b^	55.03 ^b^	24.8 ^b^	62.34 ^a^	0.185	76.09	26.64	45.27 ^a^	0.059 ^b^	53.59
YWB	②	34.45	39.63 ^c^	0.047 ^ab^	55.86 ^b^	29.44 ^a^	59.52 ^b^	0.138	75.62	27.27	41.83 ^c^	0.073 ^ab^	54.36
	③	32.47	40.33 ^c^	0.060 ^a^	56.76 ^b^	28.98 ^a^	58.42 ^b^	0.147	74.90	25.98	43.51 ^abc^	0.080 ^a^	55.06
	**0**	18.54 ^a^	49.17 ^a^	0.038 ^ab^	39.40 ^b^	31.89	50.31 ^a^	0.026	48.56 ^b^	18.07 ^a^	50.02 ^a^	0.036 ^ab^	38.82 ^b^
CBS	①	18.13 ^a^	47.81 ^ab^	0.040 ^ab^	42.06 ^a^	29.91	49.68 ^a^	0.031	51.51 ^a^	17.60 ^ab^	49.02 ^ab^	0.038 ^ab^	41.48 ^a^
	②	17.90 ^ab^	48.31 ^ab^	0.039 ^ab^	41.78 ^a^	30.82	46.97 ^b^	0.033	51.98 ^a^	17.49 ^ab^	49.23 ^ab^	0.037 ^ab^	41.28 ^a^
	③	16.10 ^b^	47.16 ^b^	0.051 ^a^	42.56 ^a^	30.91	47.06 ^b^	0.032	51.93 ^a^	15.80 ^b^	47.63 ^b^	0.049 ^a^	41.96 ^a^

^a–d^ indicate that there are significant differences of ED of the same feed in the same column (*p* < 0.05). The same applies in the following tables. 0 respectively 7 incubation time points; ① respectively 2, 16, 24, 36, 48 h; ② respectively 2, 8, 16, 24, 48 h, ③ respectively 2, 8, 16, 36, 48 h.

**Table 5:** The degradation parameters of dry matter, crude protein, and organic matter in rumen of 10 roughages (%).

		DM				CP				OM			
		a	b	c	ED	a	b	c	ED	a	b	c	ED
WS	**0**	7.30 ^a^	39.02	0.035	27.28	11.35 ^ab^	42.33	0.020	27.77	6.19 ^a^	40.02 ^a^	0.034	26.55
	①	6.13 ^ab^	37.37	0.042	27.43	9.56 ^b^	42.19	0.023	27.26	5.42 ^ab^	37.86 ^b^	0.041	26.86
	②	6.71 ^a^	38.12	0.037	27.23	9.63 ^ab^	42.89	0.022	27.21	6.13 ^a^	38.71 ^ab^	0.035	26.62
	③	6.64 ^a^	39.24	0.036	27.46	9.88 ^ab^	42.72	0.021	27.10	5.98 ^a^	39.78 ^ab^	0.035	26.83
	④	6.13 ^ab^	37.37	0.042	27.43	9.56 ^b^	42.19	0.023	27.26	5.42 ^ab^	37.86 ^b^	0.041	26.86
	⑤	5.60 ^ab^	38.71	0.043	27.93	11.55 ^a^	41.15	0.020	27.76	4.54 ^ab^	39.61 ^ab^	0.042	27.21
	⑥	4.60 ^b^	38.09	0.049	27.85	11.03 ^ab^	40.02	0.023	27.95	3.75 ^b^	38.87 ^ab^	0.047	27.11
RG	**0**	18.48	56.82	0.040	50.18	26.11 ^a^	40.04 ^b^	0.044	49.34	14.88 ^ab^	59.57 ^a^	0.039	47.63
	①	19.50	55.45	0.040	50.39	20.35 ^b^	44.50 ^a^	0.054	48.53	16.38 ^a^	58.15 ^ab^	0.037	47.84
	②	18.82	55.78	0.042	50.64	19.89 ^b^	45.29 ^a^	0.055	48.72	15.1 ^ab^	58.46 ^ab^	0.041	48.31
	③	19.48	55.03	0.040	50.34	20.59 ^b^	45.19 ^a^	0.052	48.66	16.24 ^a^	57.22 ^b^	0.039	47.79
	④	18.00	56.66	0.042	50.36	20.02 ^b^	46.25 ^a^	0.054	49.30	14.01 ^b^	59.59 ^a^	0.041	47.90
	⑤	18.95	55.97	0.039	49.99	20.47 ^b^	44.94 ^a^	0.054	48.96	15.79 ^ab^	58.61 ^ab^	0.036	47.22
	⑥	17.90	56.73	0.042	50.39	18.82 ^b^	45.88 ^a^	0.061	49.16	14.05 ^b^	59.72 ^a^	0.041	47.91
RSW	**0**	18.69 ^e^	80.71 ^a^	0.072 ^a^	66.14 ^a^	22.54 ^b^	55.64 ^a^	0.056	57.88	25.77 ^a^	54.86 ^d^	0.090 ^a^	66.79 ^a^
	①	22.16 ^d^	59.13 ^b^	0.056 ^ab^	59.97 ^b^	22.95 ^ab^	55.66 ^a^	0.051	57.48	21.51 ^de^	61.29 ^a^	0.052 ^b^	59.72 ^b^
	②	25.13 ^bc^	56.54 ^c^	0.046 ^b^	58.61 ^b^	22.90 ^b^	55.14 ^abc^	0.053	57.48	22.16 ^cde^	59.72 ^ab^	0.052 ^b^	59.40 ^b^
	③	23.47 ^cd^	57.98 ^bc^	0.051 ^b^	59.25 ^b^	22.42 ^b^	55.41 ^ab^	0.055	57.58	20.90 ^e^	61.20 ^a^	0.055 ^b^	59.91 ^b^
	④	27.70 ^a^	52.19 ^e^	0.046 ^b^	58.63 ^b^	23.7 ^ab^	53.46 ^c^	0.053	57.36	23.83 ^bc^	56.68 ^c^	0.053 ^b^	59.42 ^b^
	⑤	23.32 ^cd^	57.36 ^bc^	0.057 ^ab^	60.35 ^b^	23.71 ^ab^	54.22 ^abc^	0.052	57.52	22.89 ^cd^	59.12 ^b^	0.053 ^b^	60.07 ^b^
	⑥	25.72 ^b^	55.03 ^d^	0.050 ^b^	59.60 ^b^	24.83 ^a^	53.66 ^bc^	0.048	57.30	25.5 ^ab^	57.07 ^c^	0.046 ^b^	59.27 ^b^
BS	**0**	13.88 ^c^	31.52 ^ab^	0.084 ^ab^	36.81	11.20 ^b^	29.66	0.044	28.47	12.42	35.83 ^ab^	0.070	37.11
	①	16.62 ^a^	28.40 ^e^	0.079 ^ab^	36.96	12.02 ^ab^	30.73	0.034	27.88	13.93	34.16 ^b^	0.068	37.27
	②	14.51 ^bc^	30.62 ^bc^	0.095 ^a^	37.50	11.10 ^b^	29.74	0.045	28.42	13.05	35.04 ^ab^	0.073	37.54
	③	14.93 ^abc^	30.53 ^bc^	0.090 ^a^	37.57	10.99 ^b^	30.09	0.044	28.50	13.28	34.87 ^ab^	0.072	37.51
	④	13.90 ^c^	32.50 ^a^	0.080 ^ab^	37.24	11.11 ^b^	29.96	0.044	28.56	12.32	36.41 ^a^	0.069	37.35
	⑤	16.10 ^ab^	29.74 ^bc^	0.069 ^b^	36.55	12.53 ^ab^	30.08	0.033	27.99	13.44	35.05 ^ab^	0.064	36.98
	⑥	16.30 ^ab^	29.05 ^cd^	0.070 ^b^	36.32	13.32 ^a^	30.77	0.028	27.74	13.37	35.00 ^ab^	0.065	36.96
SS	**0**	15.75 ^ab^	47.92 ^ab^	0.087 ^bc^	50.90	20.56	55.38 ^ab^	0.075	59.54	12.87 ^a^	48.93 ^bcd^	0.085 ^b^	48.51
	①	13.83 ^b^	48.81 ^ab^	0.116 ^a^	52.26	22.45	53.01 ^c^	0.077	60.03	10.02 ^b^	50.75 ^ab^	0.114 ^a^	49.82
	②	15.83 ^a^	47.32 ^ab^	0.105 ^ab^	52.26	21.66	53.91 ^bc^	0.079	60.29	12.77 ^a^	48.62 ^cd^	0.100 ^ab^	49.73
	③	15.60 ^ab^	46.89 ^b^	0.108 ^a^	51.95	21.93	53.74 ^bc^	0.078	60.27	12.87 ^a^	47.93 ^d^	0.101 ^ab^	49.42
	④	15.69 ^ab^	48.97 ^a^	0.084 ^c^	51.38	20.49	56.11 ^a^	0.074	59.84	12.63 ^a^	50.38 ^abc^	0.082 ^b^	49.11
	⑤	15.95 ^a^	47.85 ^ab^	0.086 ^c^	50.97	21.78	54.47 ^abc^	0.070	59.43	12.27 ^a^	49.77 ^abcd^	0.086 ^b^	48.75
	⑥	15.26 ^ab^	48.82 ^ab^	0.088 ^bc^	51.19	21.74	54.27 ^abc^	0.071	59.34	11.31 ^ab^	51.02 ^a^	0.089 ^b^	49.00
CWR	**0**	14.63	43.28 ^cd^	0.024	33.24	31.16	30.34 ^a^	0.034	46.09	11.98	37.26 ^bc^	0.014	23.18
	①	14.99	44.75 ^bc^	0.021	33.13	30.77	26.66 ^b^	0.045	46.47	12.11	32.68 ^d^	0.017	23.41
	②	15.46	44.56 ^bc^	0.021	33.11	31.50	27.09 ^b^	0.037	46.19	12.53	43.01 ^a^	0.010	23.15
	③	15.06	42.71 ^d^	0.023	33.21	31.62	26.79 ^b^	0.037	46.09	12.32	37.71 ^b^	0.013	23.18
	④	15.91	45.60 ^b^	0.018	32.70	31.38	27.05 ^b^	0.037	45.99	12.48	43.12 ^a^	0.010	22.90
	⑤	15.22	45.16 ^bc^	0.020	32.85	30.40	27.12 ^b^	0.045	46.30	11.83	32.83 ^d^	0.017	23.27
	⑥	15.93	52.10 ^a^	0.015	32.62	29.76	27.62 ^b^	0.048	46.51	12.10	35.70 ^c^	0.014	23.22
RS	**0**	34.49	34.98 ^abc^	0.021	48.12	32.70 ^a^	34.97 ^ab^	0.043	52.78	32.39	35.15 ^abc^	0.022	46.38
	①	34.24	33.30 ^cd^	0.022	48.03	30.39 ^b^	36.06 ^a^	0.051	52.76	32.31	33.87 ^c^	0.022	46.35
	②	34.67	35.12 ^ab^	0.019	47.87	31.48 ^ab^	36.00 ^a^	0.043	52.35	32.73	35.84 ^ab^	0.019	46.20
	③	34.36	33.51 ^bcd^	0.022	47.98	31.65 ^ab^	35.07 ^ab^	0.044	52.10	32.40	34.38 ^bc^	0.021	46.33
	④	35.39	36.43 ^a^	0.016	47.79	33.27 ^a^	33.23 ^b^	0.043	52.42	33.12	36.61 ^a^	0.017	46.16
	⑤	34.58	33.17 ^d^	0.022	48.06	31.39 ^ab^	34.25 ^ab^	0.053	52.94	32.37	33.85 ^c^	0.022	46.39
	⑥	35.04	36.07 ^a^	0.017	47.90	30.42 ^b^	35.74 ^a^	0.057	53.39	32.86	36.20 ^ab^	0.018	46.24
OG	**0**	11.86 ^b^	67.42 ^a^	0.057	55.28	12.77 ^bc^	74.14 ^ab^	0.058	60.90	14.86 ^ab^	62.18 ^a^	0.054	54.16
	①	13.19 ^ab^	65.35 ^bc^	0.060	56.19	12.07 ^cd^	73.46 ^b^	0.068	62.39	13.71 ^b^	62.80 ^a^	0.058	54.43
	②	14.34 ^a^	64.30 ^c^	0.057	55.75	15.94 ^a^	69.97 ^d^	0.057	61.08	15.61 ^ab^	61.19 ^ab^	0.052	53.71
	③	13.89 ^a^	64.68 ^c^	0.058	55.88	14.87 ^a^	72.31 ^bc^	0.059	62.15	14.76 ^ab^	62.19 ^a^	0.054	54.03
	④	12.57 ^ab^	65.93 ^abc^	0.056	54.87	14.34 ^ab^	71.15 ^cd^	0.057	60.13	16.11 ^a^	60.08 ^b^	0.052	53.62
	⑤	11.61 ^b^	67.38 ^a^	0.058	55.30	10.51 ^d^	75.86 ^a^	0.064	61.51	13.73 ^b^	62.86 ^a^	0.058	54.49
	⑥	12.43 ^ab^	66.58 ^ab^	0.056	55.06	11.78 ^cd^	74.07 ^ab^	0.062	61.00	14.86 ^ab^	61.52 ^ab^	0.055	54.10
CS	**0**	35.33 ^b^	36.29 ^a^	0.053	59.63 ^ab^	36.53	37.58	0.080	64.86	34.35 ^a^	37.24 ^b^	0.049 ^c^	59.56 ^ab^
	①	37.52 ^a^	33.61 ^b^	0.051	60.04 ^a^	36.23	37.93	0.079	65.00	35.87 ^a^	35.25 ^c^	0.054 ^ab^	59.92 ^a^
	②	37.61 ^a^	33.25 ^b^	0.052	59.96 ^a^	37.25	36.87	0.074	64.71	26.09 ^b^	43.95 ^a^	0.071 ^a^	58.48 ^abc^
	③	37.18 ^ab^	34.35 ^b^	0.052	60.35 ^a^	37.02	36.99	0.075	64.71	35.75 ^a^	35.79 ^bc^	0.055 ^ab^	60.11 ^a^
	④	35.56 ^b^	36.13 ^a^	0.051	57.87 ^bc^	37.38	36.19	0.077	63.06	34.87 ^a^	36.54 ^bc^	0.052 ^ab^	57.59 ^c^
	⑤	35.70 ^ab^	36.39 ^a^	0.049	57.88 ^bc^	36.23	37.71	0.081	63.42	34.49 ^a^	37.43 ^b^	0.052 ^ab^	57.83 ^bc^
	⑥	36.97 ^ab^	34.90 ^b^	0.044	57.32 ^c^	36.37	37.69	0.080	63.46	35.95 ^a^	35.68 ^bc^	0.046 ^c^	57.21 ^c^
TP	**0**	11.83 ^b^	82.86 ^abc^	0.043	50.11 ^d^	18.61 ^a^	78.66 ^d^	0.064 ^a^	62.55 ^b^	21.71 ^a^	76.52 ^d^	0.074 ^a^	67.14 ^b^
	①	15.55 ^a^	84.38 ^a^	0.046	69.82 ^a^	15.71 ^b^	83.35 ^bc^	0.036 ^b^	64.60 ^a^	10.47 ^d^	89.13 ^a^	0.049 ^b^	69.37 ^a^
	②	17.06 ^a^	82.27 ^bc^	0.045	69.81 ^a^	14.41 ^b^	85.34 ^a^	0.039 ^b^	66.38 ^a^	16.34 ^c^	83.32 ^b^	0.046 ^b^	69.93 ^a^
	③	15.96 ^a^	84.01 ^ab^	0.046	69.99 ^a^	15.08 ^b^	82.10 ^c^	0.040 ^b^	65.47 ^a^	18.73 ^b^	80.20 ^c^	0.045 ^b^	70.23 ^a^
	④	16.14 ^a^	81.86 ^c^	0.041	62.40 ^b^	15.42 ^b^	81.75 ^c^	0.038 ^b^	60.39 ^c^	18.37 ^b^	80.30 ^c^	0.044 ^b^	64.96 ^c^
	⑤	15.44 ^a^	82.35 ^bc^	0.041	61.83 ^bc^	15.64 ^b^	83.17 ^bc^	0.037 ^b^	60.35 ^c^	17.82 ^bc^	81.94 ^bc^	0.043 ^b^	65.27 ^c^
	⑥	15.23 ^a^	84.57 ^a^	0.036	60.17 ^c^	14.97 ^b^	84.42 ^ab^	0.036 ^b^	60.06 ^c^	18.52 ^b^	81.24 ^c^	0.041 ^b^	64.28 ^c^

^a–e^ indicate that there are significant differences of ED of the same feed in the same column (*p* < 0.05). 0 respectively 7 incubation time points; ① respectively 4, 16, 36, 48, 72 h; ② respectively 4, 16, 24, 48, 72 h; ③ respectively 4, 16, 24, 36, 72 h; ④ respectively 4, 8, 16, 24, 72 h; ⑤ respectively 4, 8, 16, 36, 72 h; ⑥ respectively 4, 8, 16, 48, 72 h.

## Data Availability

Publicly available data sets were analyzed in this study, and these have been referenced in the manuscript.

## Abbreviations

RSM (rape seed meal); CSM (cotton seed meal); CGM (corn gluten meal); ES (expanded soybean); DDGS (distillers’ dried grains with soluble); SOM (soybean meal); BR (brown rice); BY (barley); WT (wheat); HS (Heilongjiang sorghum); HY9 (Heilongjiang corn Heyu 9); GWC (Guizhou white corn); YC (Yunnan corn); YWB (Yunnan wheat bran); CBS (spouting corn bran); WS (wheat straw); RG (rye grass); RSW (radish straw); BS (beckmannia syzigachne); SS (soybean stem); CWR (Chinese wild rye); RS (rye straw); OG (orchard grass); CS (corn silage); TP (turnip root).

## References

[1] AFRC (1992). Nutritive requirements of ruminant animals: Protein. Nutr. Abstr. Rev..

[2] Madsen J., Hvelplund T. (1994). Prediction of in-situ protein degradability in the rumen-results of a European ringtest. Livest. Prod. Sci..

[3] Ørskov E.R. (1982). Protein Nutrition in Ruminants.

[4] Lindberg J.E. (1985). Estimation of rumen degradability of feed proteins with the in sacco technique and various in vitro methods. A review. Acta Agric. Scand. Sect. A-Anim. Sci. Suppl..

[5] Michalet-Doreau B., Cerneau P. (1991). Influence of foodstuff partice-size on in situ degradation of nitrogen in the rumen. Anim. Feed Sci. Technol..

[6] Vanzant E.S., Cochran R.C., Titgemeyer E.C. (1998). Standardization of in situ techniques for ruminant feedstuff evaluation. J. Anim. Sci..

[7] Seradj A.R., Morazán Nuñez H.J., Fondevila M., Liang J.B., de la Fuente Oliver G., Balcells Terés J. (2019). In Vitro and In Situ Degradation Characteristics and Rumen Fermentation Products of Moringa oleifera Harvested at Three Different Ages. Trop. Anim. Sci. J..

[8] Tao L., Zhang L.-X., -Tu Y., Zhang N.-F., Si B.-W., Ma T., Diao Q.-Y. (2016). Improving the in situ ruminal degradability of maize stalk using fungal inoculants in dorper × thin-tailed han crossbred ewes. Small Ruminant Res..

[9] Ghorbani B., Teimouri Y.A., Jafari S.A. (2018). Effects of sesame meal on intake, digestibility, rumen characteristics, chewing activity and growth of lambs. S. Afr. J. Anim. Sci..

[10] Diao X., Dang S., Liu S., Jing L., Wang Y., Zhang W. (2020). Determination of the appropriate ratio of sample size to nylon bag area for in situ nylon bag technique evaluation of rumen digestibility of feedstuffs in sheep. Livest. Sci..

[11] Rjiba-Ktita S., Chermiti A., Bodas R., France J., López S. (2017). Aquatic plants and macroalgae as potential feed ingredients in ruminant diets. J. Appl. Phycol..

[12] Harahap M.A., Nuswantara L.K., Pangestu E., Wahyono F., Achmadi J. (2018). Nitrogen degradation of the limestone-urea mixtures in the rumen of goats. J. Indones. Trop. Anim. Agric..

[13] Palangi V., Macit M. (2019). In situ crude protein and dry matter ruminal degradability of heat-treated barley. Rev. Med. Vét..

[14] Vahidi M.F., Gharechahi J., Behmanesh M., Ding X.Z., Han J.L., Salekdeh G.H. (2021). Diversity of microbes colonizing forages of varying lignocellulose properties in the sheep rumen. PeerJ.

[15] Jiang B., Zhou Y., Wang T., Li F. (2020). Nutritive value and ruminal degradation of seven Chinese herbs as forage for Tan sheep. Bioengineered.

[16] Passetti R.A., Passetti L.C., Gruninger R.J., Ribeiro G.O., Milani M.R.M., Prado I.N., McAllister T.A. (2020). Effect of ammonia fibre expansion (AFEX) treatment of rice straw on in situ digestibility, microbial colonization, acetamide levels and growth performance of lambs. Anim. Feed Sci. Technol..

[17] Association of Official Analytical Chemists (AOAC) (1997). Official Methods of Analysis.

[18] Van Soest P.V., Robertson J.B., Lewis B.A. (1991). Methods for dietary fiber, neutral detergent fiber, and nonstarch polysaccharides in relation to animal nutrition. J. Dairy Sci..

[19] Mehrez A.Z., Ørskov E.R., McDonald I. (1977). Rates of rumen fermentation in relation to ammonia concentration. Br. J. Nutr..

[20] Lopez S., Khalil M.S. (2012). In situ degradability of soyabean meal treated with Acacia saligna and Atriplex halimus extracts in sheep. J. Anim. Feed Sci..

[21] Ørskov E.R., Mcdonald I. (1979). The estimation of protein degradability in the rumen from incubation measurements weighted according to rate of passage. J. Agric. Sci..

[22] Promkot C., Wanapat M., Rowlinson P. (2007). Estimation of ruminal degradation and intestinal digestion of tropical protein resources using the nylon bag technique and the three-step in vitro procedure in dairy cattle on rice straw diets. Asian Australas. J. Anim. Sci..

[23] Messman M.A., Weiss W.P., Koch M.E. (1994). Changes in total and individual proteins during drying, ensiling, and ruminal fermentation of forages. J. Dairy Sci..

